# Suffering, authenticity, and physician assisted suicide

**DOI:** 10.1007/s11019-019-09929-z

**Published:** 2020-07-13

**Authors:** R. Ahlzen

**Affiliations:** Karlstads Universitet, Karlstads, Sweden

Neither will I administer a poison to

anybody when asked to do so, nor will

I suggest such a course

The Hippocratic Oath.

## Introduction

These lines in the Hippocratic Oath seem clear enough. The oath prohibits physicians to facilitate for their ill patients to take their lives. This conclusion has been questioned, suggesting that what is meant is a warning for the administration of drugs with serious side effects. However, evidence supports that the Hippocratic physicians did not participate in what we call assisted suicide (van Hooff 2014).

There may, now as in the Greek antique, be degrees of suffering that make life very hard to live, and which may occasionally turn death from being a threat into being a last resort. Such suffering may be ameliorated or even made disappear through medical intervention, or by the appearance of some strongly meaning-giving person or insight. In Lev Tolstoy’s *The Death of Ivan Iljitj*, the terminally ill protagonist screams during several days out of pure desperation, when, finally, a young man appears and with his words and his presence manages to give peace to the tormented soul (Tolstoy 1887). If such endeavors are unsuccessful persons may die in agony and despair.

The rise of medical ethics in the 70’s and 80’s was, as could be expected, followed by a vitalized debate on end of life decisions. New techniques for life sustaining treatment and better remedies for chronic disorders gave rise to expectations of a longer life with less suffering. New groups of professionals appeared at the bedside of the seriously ill person, demanding influence on decisions concerning life and death matters (Rothman 1991). But in spite of improved palliative care, suffering at the end of life was still there, and however strong analgesic drugs that were used, there remained some persons who suffered at the end of their lives.

The last two or three decades have seen a gradual shift of attitudes on end of life issues. Value surveys, like World Value Survey, from Western Europe and North America disclose a pattern of a slow shift towards more permissive attitudes concerning the active shortening of seriously ill persons’ lives under certain circumstances (Cohen et al. 2013). The tendency over time is so clear that the conclusion can hardly be questioned. This has been followed by a legislative shift in country after country, making the right to some kind of medically assisted shortening of life for seriously ill persons on their request more common. It is, however, important to notice that there are several models for this, and that these differ substantially concerning their ethical foundation. “The Benelux model”, the far-reaching euthanasia practice of the Netherlands and Belgium, is, many argue, ethically distinct from the limited and restrictive practice of physician assisted suicide (PAS) in, for example, the American state of Oregon.

In the intense debate about different practices of euthanasia the ethical principle of autonomy has played a major role. It has been proposed that the right to decide about the moment of one’s death is a consequence of the right to take decisions concerning one’s body and what the health care system should, or should not, do with it. “My death is mine” may be a summary of this attitude, and if this includes the wish to be helped to die before the disease itself takes life away, then be it so and physicians have a duty to help in that. Against this has been argued while there is a right to self-determination in the health care system, this does certainly not include the right to demand of someone, in this case a physician, to kill you or to give you the means to end your own life.

The same argument can be made, instead relying on the concepts mercy or dignity. When a person risks losing, or has already lost, his or her dignity, and this process is judged irreversible, it is an act of mercy following the best ethical traditions of medicine to shorten that suffering. In line with this, it is emphasized that the prolonging of life, the fight against death, has never been and should never be a task that triumphs over the duty to relieve suffering, and if this cannot be done in any other way, then the shortening of life is ethically acceptable, or even mandatory. Again, arguments against this position assert that dignity can be restored, that human dignity may remain also in bodily decay and dependency and that assuming terminal illness to be undignified may become a self-fulfilling prophecy.

Shortening of life to provide escape from suffering is, not surprisingly, nothing entirely new, as also seen from the quotation above from the Hippocratic oath. A well-known example is the death of Sigmund Freud, who died in London in 1939. The year before, he had fled from the Nazi occupied Austria in company with his friend and physician Max Schur and his daughter Anna. Freud was seriously, probably terminally, ill with an epithelioma of the hard palatine. He had been operated many times and was now inoperable. Only severe and intensified suffering remained. He then asked his physician friend to give him high doses of morphine so that he slept deeply until death came. Schur did that, using the means that he had at hand, after Freud had taken farewell of those of his family who were also there. On the 3rd day, after two more injections, Freud peacefully died (Gay 1988, pp. 648–651). Freud was clearly in pain but it seems that mental decline and bodily decay were the major sources of his suffering. Thirty years earlier, he had written in a letter to a friend about what to do when “…thoughts fail and words will not come?”. Freud admitted that he could not avoid to

…tremor before that possibility. That is why, with all the resignation before destiny that suits an honest man, I have one wholly secret entreaty: only no invalidism, no paralysis of one’s powers through bodily misery. Let us die in harness, as King Macbeth says (p. 651).

Freud asked for what is now called terminal sedation, defined as an intentional lowering of a terminally ill person’s consciousness in order to relieve pain, but he could just as well had asked Schur for a number of pills that he knew would be enough to let death come. The ethical difference between these two options is much discussed and will not be dealt with further here. What we will explore is how a suffering like Freud’s can be understood, and whether the notion of *authenticity* may help us overcome some of the limitations of an argument based primarily on the right to self-determination.

## Physician assisted suicide

Before approaching the concept of authenticity, some conceptual clarity must be achieved. Physician assisted suicide, PAS, is by some seen as one distinct model of euthanasia. It is, however, not an immediate active intervention of a physician that kills the patient, it is the action (taking a drug) of the ill person himself or herself. The physician *makes possible* an action that ends life, and—the proponents insist—cannot be said to be ultimately responsible for it. Definitions are crucial here. Often euthanasia is used only to designate a physician’s momentary and intentional ending of a seriously ill person´s life on his or her request. This would exclude PAS. Physician assisted suicide may be defined as: “A physician intentionally helping a person to terminate his/her life by providing drugs for self-administration, at that person’s voluntary and competent request” (Radbruch et al. 2016).

In Oregon around 130 terminally ill persons take their life yearly with the help of physician prescribed drugs. Considerably more patients (around 200) had been afforded the option, but in the end, not all use it. The role of the physician extends beyond the prescription. S(he) is also supposed to have ruled out depression as a cause of the ill person´s suffering. The patient must be judged competent by two physicians and this must be done with a time interval of at least 2 weeks. Furthermore, a prognosis must be established from best medical judgement, that life expectancy for the ill person is less than 6 months (Ganzini et al. 2001).

In Switzerland, like for example for *Dignitas* in Zurich, this latter requirement does not exist. Persons with chronic disorders and rather long estimated remaining life time but deep suffering may be allowed to receive PAS. Common to both models is that the ill person must suffer deeply, and that no relief for this is in sight. Both include the evaluation of decision-making competency by two independent physicians.

The critique against PAS has developed along at least three interrelated lines. One is that prognosis for both remaining life time and the possibility of new options appearing for remedy and alleviation are by necessity uncertain factors. This critique implies that persons who could have received support to help them want to live, or who might have passed into a better situation, instead are invited to take their lives. A second objection is that if, for example, PAS is allowed, palliative care will be less prioritized and thereby persons who could actually have been helped to live decent lives will not be so. A third argument proposes that PAS violates the central medical ethical principle of respect for life. Doctors should not take lives, and should not help persons to take their lives. They should save lives, and make lives more worth living. To this is often linked a slippery slope argument, claiming that allowing PAS will pave the way for other, even more ethically dubious, measures from physicians to shorten their patients’ lives. Ill persons, it is said, will ask for this as they interpret the possibility to choose death also as a demand not to be a burden on those alive and on their society’s health care costs.

Some of these arguments have a factual component, which can be empirically assessed. For example, no tendency for palliative care to be less prioritized is seen in for example Oregon, though it cannot be established without doubt that palliative care in Oregon would not be even better off if PAS had not been allowed. There has been only a slight increase in the number of PAS during the 21 years that the law has existed, and the level is still on less than 0.5% of the total number of deaths (Ganzini 2016). More difficult to judge is the possible impact on ill persons’ view of themselves. We do not know whether the possibility to choose PAS by those seriously ill is experienced as something that makes suffering easier to bear, as the ill person knows that there may be a self-controlled end—or whether some persons may see the option to use PAS as more than just an option but rather as a duty, in order not to be a burden on family or on the health care system. The very fact that this may be the case constitutes a serious challenge to the ethics of PAS.

## Autonomy in health care

In the context of health care, autonomy is usually interpreted as the right to self-determination, and when this is not possible, to co-determination. Autonomy in health care is seen both as a capacity, linked to a person’s cognitive and emotional status, and as a right among other rights in a society that has increasingly acknowledged individuals as self-governing units within a social-political framework. Medical ethics has consequently during the last two or three decades largely focused on issues around autonomy—its limits, its preconditions, its expressions. To judge decision-making capacity has been seen as one of the central capacities involved in clinical judgement (Ahlzén 2010).

The patient has an absolute right to say no to a suggested treatment, in case (s)he is competent, but there is no corresponding right to demand a certain intervention. If this restriction is accepted, the principle of autonomy can, as noted above, hardly be recruited as sole support for the right to receive help to PAS. A person cannot require a physician to initiate a treatment which (s)he judges to be medically dangerous and hence unethical. If, however, the same physician reaches the conclusion that beneficence and mercy dictate that the terminally ill person is given this possibility, and if such an action is legal—then the ethics will rely on whether this *is* really a beneficent act and *if* the ill person expresses his or her true self, that is: if the choice stems out of the persons central values, her deepest orientation in life.

As we have seen, where PAS has been legally accepted it has without exception been tied to an evaluation of competency. In practice, this has meant that clinically relevant depression as far as possible is ruled out, and that no serious impairment of cognitive capacities can be discerned. Depression is then seen as a condition where an individual, due to a pathological process changing his or her way of looking at herself and the world (loss of hope, loss of future, inertia, feelings of guilt and loss of self-esteem), is obstructed from using those combined emotional and cognitive capacities that are the foundation of autonomy. If there is no clinically recognizable depression and if no other cognitively disturbing process can be identified, the patient is judged competent. But questions remain about the origins of the will to die, the congruence between this wish and the person’s identity, “true self”.

The concept competency is clearly linked to autonomy. But it may be argued that a decision can be autonomous, that is: be made by a competent person, for example not suffering from a serious mental disorder, but still not be authentic. If so, this opens a possibility to take the discussion in the case of PAS one step further. It is, in this case, not only necessary to attempt to judge whether an individual is autonomous in the usual, “emocognitive”, meaning of the word—reasonably rational and emotionally stable—but (s)he should also be sufficiently authentic, in some meaning of this concept. If such a need for a qualification of the “autonomy condition” is accepted, we then face the challenge to judge whether exactly the concept authenticity is a suitable candidate for this.

## Authenticity

To bring in the concept authenticity into this discussion is to invite ambiguity. Few concepts have been so radically differently interpreted as authenticity. Is this difficulty so deep-going that it makes the concept unsuitable in this context?

In philosophical encyclopedias, the usual subdivision includes a strong emphasis on the existentialist use of the concept, which in turn is heterogeneous, for example the obvious differences between Kierkegaard’s and Heidegger’s use of the notion. One will also encounter psychological and religious interpretations. One consequence of this array of suggestions on the meaning of authenticity is that the critique against the “the cult of authenticity” seems to strike only certain interpretations, and not necessarily others. Social historian Christopher Lasch, for example, associates the cult of authenticity with an increased self-indulgence, with the growth of a narcissistic personality type in Western societies (Lasch 1982). In a similar vein, political theorist Harold Bloom maintains that the wish to be authentic leads to self-centeredness and makes the minds “narrower and flatter” (Bloom 1988, p. 61). But if authenticity is not seen as referring to a self that is an atomistic self-governing unit, maximizing its pleasure irrespective of consequences for others, as these two authors imply, then authenticity is not necessarily connected to egocentricity and narcissism. On the contrary, it may be connected to social values and a motivation to grow in maturity and knowledge of the world, while remaining true to oneself.

Charles Taylor is the contemporary philosopher who has done most to reinvigorate authenticity as a tool for understanding Western culture in the late twentieth and early twenty-first century. His *The Ethics of Authenticity* came in 1991, at a time when this debate had gained considerable momentum. Taylor describes the rise of authenticity as a consequence of the ongoing increase of individualism, and also as a reaction against the “disenchantment” of the world, that is as an attempt to counteract what was seen as an instrumentalized view of society and the human condition. But the reaction was risky, and “… new modes of dependence arise among people who are striving to be themselves, and beyond this new forms of dependence…” (p. 15). Emphasizing autonomy, a notion seen as an expression of authenticity, also has its dangers: “…the notion of self-determining freedom, pushed to its limits, doesn’t recognize any boundaries….” (p. 68). Taylor agrees with the critique against a widely spread relativism connected to this way of looking at the authentic self. However, he does not want to do away with the concept altogether but rather reinterpret it. “Rather, we face a continuous struggle to realize higher and fuller modes of authenticity against the resistance of the flatter and shallower forms.” (p. 94)

Taylor hence wants the concept to be reinterpreted in a far less self-indulgent way than was usually done at the time. Authentic is a person who by being true to himself or herself also actualizes the best in her human nature, who connects to sources outside herself, who strives to identify a common good and work for it. It is not difficult to discern a connection to Heidegger where an authentic life form is one where authenticity has to do with *being* a person of a particular sort, with a sense of wholeness, with being connected to the ongoing life. There is no pregiven, “true”, inner nature to connect to here, rather an ongoing way of being in one’s life which presupposes a set of capacities, values, orientations and dispositions. (Stanford Encyclopedia of Philosophy 2019) When these are seriously threatened or destroyed, the possibility of an authentic life is no longer there. The individual is, in the deepest sense of the word, no longer himself or herself.

For the sake of this discussion of PAS, I suggest that the ethic of PAS depends, among several circumstances, on whether the ill person expresses an authentic will. The will is authentic only if it is deeply connected to values, orientations, emotions, motivations and dispositions that the person holds in a more or less reflected way, and identifies as his or her *own,* as part of and expressed in an ongoing flow of life-events. Self-deceit, illusions, delusions, self-destructiveness, and ignorance, sometimes but not always parts of cognitive decline and grave mood disturbances, threaten authenticity. Being authentic is a wider notion than being competent. A decision can be competent but still not necessarily authentic. This fact raises demands on those who deal with persons who want help to end their lives due to intractable suffering. Judging authenticity is extremely difficult and hence this may be both an argument for, and against, the legalization of PAS. It must be kept in mind that several of the arguments against PAS remain even if the will to receive it is judged to be authentic.

## Suffering and authenticity

Eric Cassell has suggested that we define suffering as”…the state of severe distress associated with events that threaten the intactness of the person” (Cassell (1991), p. 33). Why is there a threat to an individual’s “intactness” associated with suffering? I suggest it is because being intact, in Cassell’s sense, makes it possible to be authentic. It is the very precondition for realizing one’s core values, evaluating one´s inclinations, scrutinizing one’s very basis for orientation in life—in effect, *for being true to oneself*. Lost intactness means lost core functions, lost abilities to attain central goals in life, loss of that “rhythm” in everyday life that characterizes the authentic life. Cassell discerns several strategies to reduce suffering—living in the present, denial, developing indifference, flexibility—but neither of these necessarily work very successfully in the face of suffering at the very end of life.

Fredrik Svenaeus’ position is close to Cassell’s. He suggests we look upon suffering as “…an alienating mood overcoming a person and engaging her in a struggle to remain at home in the face of loss of meaning and orientation in life.” (Svenaeus 2017, p. 33) He finds it important to evaluate the degree of severity. Not all negative moods entail suffering. Suffering appears when the embodied “being-in-the-world” of a person is alien, “unhomelike”. Such a mood “… affects the entire existence of the ill person” (p. 31). With no meaningful orientation and with core life values obstructed, life is inauthentic, and suffering will result.

What makes a life worth living? Svenaeus deals with this question, inspired by Charles Taylor who in his book *Sources of the Self* describes how our selves, our identities, rest on what he calls *strong evaluations* (Taylor 1989, p. 4). Such strong evaluations are like the supporting pillars for our sense of meaningfulness, the necessary preconditions for finding life worth living. If terminal disease strikes against these, why does a person want to go on the months until the liberating death comes? Because of a hope that things will come to look differently in a short time? But time is already short. Serious life-threatening disease with a prognosis for less than 6 months more to live—the criteria for PAS in Oregon—is not. Long time for reorientation. But then again, is not the above description of obstructed “strong evaluations” a description of depression, a condition at least in principle possible to treat—and one of the exclusion criteria for PAS? This could be the case but not necessarily. Not all experiences of inauthenticity are secondary to depression, but of course loss of authenticity in life- threatening disease may prompt depressive symptoms.

## Authenticity and PAS

Being authentic, as we noted, means being “at home” in that rhythm of life, that uniquely personal way of relating to the world, that is deeply embedded in a person’s values, desires, inclinations, beliefs. We also draw the conclusion that major depressive disorder is not compatible with being authentic, and neither are other serious psychiatric disorders or later stages of dementia. It is characteristic of such states that close relatives do not recognize the ill person as the one (s)he “really is”. “He is another person”, is a common remark, or, in such cases where there has been a recovery: “I was not myself”. Such ways of being inauthentic are largely due to pathophysiological processes affecting the brain. Patients even with advance stages of dementia may have good lives, though they are clearly inauthentic, again reminding us that inauthenticity does not in itself equal loss of life quality. Inauthenticity can also result from other debilitating diseases, like for example late stage cancer, ALS and grave forms of MS, certain progressive neuromuscular disorders, serious trauma with severe loss, which profoundly change a person’s possibility to live an authentic life characterized by that “rhythm”, that resonance, that was there before the disease.

It may seem strange that, if we accept depression as an obvious source of inauthenticity, this condition should still serve as a counter-indication for PAS. But depressions can be often be cured and most of them have a limited natural span also without treatment. Authenticity, and with this the will to go on living, may then return—albeit in a more or less transformed form. It is different with an inauthenticity that is the result not primarily of a depressive mood, but of fundamental and irreversible losses in life, like the capacity to move reasonably freely without pain, to control basic bodily functions, manage the intake of food and liquid, to conduct everyday cognitive operations, to recall and react to memories shared with other persons. They may find new ways to compensate for and find meaning in. this—or they may not.

A wish for PAS should, according to legislation in countries where such help is allowed, be, in the formal sense, competent. If PAS is accepted—should it, in addition to that, be up to the physician(s) to judge the patient’s degree of authenticity? Such a judgement demands that the physician has a reasonably good knowledge of the ill person, which is probably seldom the case. In the Netherlands, where most cases of euthanasia are performed by GP:s with a knowledge of the ill person over many years, this may sometimes be possible. In Sweden, as a contrast, few patients have a primary care physician since a long time, if at all, and here the evaluation whether a person’s wish for help to end life by an action of his or her own faces a greater challenge. Those who advocate PAS must, I contend, make plausible that the physicians who perform this have the knowledge, the interest and the experience to judge how authentic the wish of the ill person is, and not just formal competency.

## A suffering beekeeper

The novel by Lars Gustafsson, *The Death of a Beekeeper,* appeared in 1978 as the last in a series of five novels, *The Cracks in the Wall.* It has been extensively dealt with by Bondevik et al. (2016) as an example of how literature may capture the ambiguity and complexity of a person´s reaction to very serious bodily symptoms. This analysis will not be repeated here, but the novel is also an invitation to reflect on what it means to have, or not to have, an authentic life, when this same life is seriously threatened by illness. The novel is a reminder of how difficult it may be to capture a person’s inner world and to judge the degree of authentic sense of life that (s)he experiences. It is to be remembered, of course, that this fictive beekeeper is as unique in his reaction in his struggle for meaning and coherence as any other person who would suffer increasing distortion of the patterns of everyday life.

In fragmented notes, the reader is presented with glimpses of Lars Westin’s life. He now lives alone in a rural little house with his dog, obviously subsisting on the modest earnings from his bee hives. His life narrative is offered us as fragments and so are his reactions to the increasingly alarming symptoms. While following him in the mundane matters of everyday life, we can see how these are transformed in an alien way by the illness, which is increasingly invasive into his thoughts and dreams, and see how he struggles to make some sense of what is going on. Clearly enough, his life becomes increasingly inauthentic. The intactness, his sense of life as a whole, breaks down. A passage illustrates this:

What I experience is total dissolution, total confusion. Up to now, I never really grasped that the possibility of experiencing ourselves as something clearly defined, ordered, as a human self, depends on the possibility of a future. The foundation of the entire concept of the self is that it will continue to exist tomorrow. (p. 79)

Who is really Lars Westin? He strikes the reader as an enigmatic person. The scattered notes in the left books show him as a man who maybe never really has taken place in his own life. He studies, he marries (the wrong woman), he divorces (seemingly without any sentiments), he leaves his job as a teacher to live lonely with his dog and his beehives. Is his life inauthentic or is it rather the opposite? Has the beekeeper really stepped out of an increasingly inauthentic social life, to *really become more of himself?* If so, how shall we understand the fact that when the letter with the final verdict on his disease finally comes, after months of investigations, he burns it, in an oddly indifferent and distanced way?

It is clear that inauthenticity often makes life less worth living. However, this is not necessarily the case. One may imagine an inauthentic life, a person who loses contact with who (s)he really is, but stills feels fine, enjoys life. Far reaching self-betrayal may act this way, but in the longer run, inauthenticity probably tends to erode also life quality.

When the future is seriously threatened, the self is under attack. Any diagnosis, or in the beekeeper’s case strong suspicion, of a life-threatening disorder will carry such a challenge. The reader of the novel is invited to see how the fragments (s)he is presented of the beekeeper’s life narrative offer clues to an understanding of his reactions. Finally, the novel leaves us with a question: Was his decision not to undergo treatment for what we know was a cancer of the spleen really an authentic decision? Did it mirror his “true self”, reflect the core values of his life? If he had accepted treatment, and thereby received some more time, would this have deeply changed his self-understanding? If a relapse then had occurred, and all hope for curative treatment was gone, would he then have asked for PAS? Arthur Frank calls the constructive answer to a break of life narrative due to the experience of a life-threatening disease—a “quest story” (Frank 1995). Would there have been such a story at hand for the beekeeper during his last months alive?

## Conclusion

I have argued that loss of authenticity often accompanies serious disorders, particularly debilitating such. Such loss, and also the threat of it, often but not always causes suffering. If the disease is life-threatening the time for reorientation is often short. A person may want death to come before the destruction of his or her self has been completed. He or she may ask for help to end his life before the disease does. Should such a wish be respected?

The concepts authentic and authenticity may seem promising in connection to judgement of such a demand. At the same time, it is obvious that authenticity is a deeply ambiguous concept, and judging what is an authentic or inauthentic life may be filled with pitfalls. The beekeeper in Gustafsson’s novel illustrates this. The reader is left in uncertainty about his sense of self, his core personality, his basic values. This uncertainty adds depth to the reflection on what an authentic decision may be in the case of PAS. It must also be noted that another aspect of the novel involves the contemporary society from which Westin has tried to withdraw. In glimpses we are shown a society of lies and alienation, and of diminishing sense of direction and meaning. In this way, the possible loss of identity and authenticity in the beekeeper’s life is paralleled by the same process in the surrounding society.

Autonomy and authenticity are interdependent concepts but ought to be kept apart. Autonomy includes a number of cognitive capacities, and also demands a basic degree of emotional stability. Authenticity can be intact while autonomy is reduced, and the other way around. Still, any legislation on PAS must include criteria concerning competency. Loss of autonomy due to serious cognitive disturbances should be a relative obstacle to PAS.

We need to develop a richer and fuller understanding of what the conditions are for leading an authentic life in serious disease. I suggest that the discussion on the ethics of PAS must not restrict itself to a discussion on the meaning and limits of autonomy, but to include also considerations on the relation between autonomy and authenticity.
